# Efficacy of nitrous oxide in adults undergoing puncture biopsy: A systematic review and meta-analysis of randomized controlled trials

**DOI:** 10.1371/journal.pone.0286713

**Published:** 2023-06-06

**Authors:** Ziyang Wang, Fei Wang, Yihui Xing, Xiaochen Jiang, Zhiguo Ding, Yuxiang Li, Lu Tang

**Affiliations:** 1 School of Nursing, Weifang Medical University, Weifang, China; 2 Department of Stomatology, The 960^th^ Hospital of People’s Liberation Army of China (PLA), Jinan, China; 3 Department of Anesthesiology, The 960^th^ Hospital of People’s Liberation Army of China (PLA), Jinan, China; 4 Department of Hepatopancreatobiliary Surgery, Qingdao Municipal Hospital, Qingdao, China; 5 Ningxia Medical University, Yinchuan, China; Sapienza University of Rome: Universita degli Studi di Roma La Sapienza, ITALY

## Abstract

**Background:**

Nitrous oxide (N_2_O) with rapid analgesic effect is often used to relieve pain induced by diagnostic procedures. This review was conducted to evaluate the efficacy and safety of N_2_O in patients undergoing puncture biopsy.

**Methods:**

We systematically searched PubMed, Embase, the Cochrane Library, Web of Science, Scopus and the ClinicalTrials.gov up to March, 2022. Randomized controlled trials (RCTs) were included if they investigated the effect of N_2_O in adults undergoing puncture biopsy. The primary outcome was pain score. Secondary outcomes included anxiety score, patient satisfaction and side effects.

**Results:**

Twelve RCTs with 1070 patients were included in the qualitative review, of which eleven RCTs were included in the meta-analysis. Pooled analysis suggested that compared with the controls (placebo, lidocaine and midazolam), N_2_O had better analgesic effect (MD -1.12, 95% CI -2.12 to -0.13, P = 0.03; I^2^ = 94%). In addition, N_2_O significantly alleviated patient anxiety (MD = -1.79, 95% CI -2.41 to -1.18, P<0.00001; I^2^ = 0%) and improved patient satisfaction (MD 1.81, 95% CI 0.11 to 3.50, P = 0.04; I^2^ = 92%). There was no significant difference regrading the risk of nausea (RR 2.56; 95% CI 0.70 to 9.31, P = 0.15; I^2^ = 0%), headache (RR 0.62, 95% CI 0.17 to 2.33, P = 0.48; I^2^ = 46%), dizziness (RR 1.80, 95% CI 0.63 to 5.13, P = 0.27; I^2^ = 0%) or euphoria (RR 2.67, 95% CI 0.81 to 8.79, P = 0.11; I^2^ = 8%) between the N_2_O group and the control group.

**Conclusion:**

The present review suggested that N_2_O might be effective for pain management in patients undergoing puncture biopsy.

## Introduction

Puncture biopsy is the main way to gain tumor tissue or cell sample for histopathological diagnosis [1], such as percutaneous liver biopsy (PLB), lumbar puncture (LP), bone marrow puncture and transrectal ultrasound-guided prostate biopsy (TUSPB). It can provide strong proof for identification of benign and malignant tumors and selection of the appropriate remedy scheme [2–6]. Puncture biopsy is painful and unpleasant [7–10], and the anxiety and fear also increase the perception of pain, which may hinder the patient compliance with future biopsy.

Nitrous oxide (N_2_O) is an inhalational agent with analgesic, sedative and anti-anxiety properties [11]. It has the advantages of simple handling, fast onset and few adverse reactions and has been shown to have good analgesic effects in various settings such as dental, emergency treatment, obstetrics and pediatrics [12–16]. Recently, several randomized controlled trials (RCTs) investigated the effects of N_2_O regarding pain management in patients undergoing puncture biopsy and no consistent conclusion was made. Thus, we conducted a systematic review and meta-analysis of RCTs to assess the efficacy and safety of N_2_O in patients undergoing puncture biopsy.

## Materials and methods

This systematic review and meta-analysis was conducted following the current recommendations of the Cochrane Collaboration and was reported according to the guidelines of the Preferred Reporting Items for Systematic Reviews and Meta-Analyses [17]. The protocol was registered on PROSPERO (registration number: CRD42022321185).

### Literature search and eligibility criteria

Relevant articles were identified by searching PubMed, Embase, the Cochrane Library, Web of Science, Scopus and the ClinicalTrials.gov (up to March, 2022) without language restriction. Electronic searches were conducted using the Exploded Medical Subject Headings and appropriate corresponding keywords: “nitrous oxide”, “Entonox”, “N_2_O”, “puncture”, ‘‘biopsy” and ‘‘puncture biopsy”. We also inspected the reference lists of studies identified by the previous searches for additional studies eligible for inclusion. Two authors independently assessed the eligibility of all studies identified in the initial research. Studies meeting the following criteria were included: (1) Population: adults undergoing puncture biopsy; (2) Intervention: N_2_O inhalation; (3) Comparison: placebo or other analgesic methods; (4) Outcome: at least one of the following endpoints: pain score, anxiety score, patient satisfaction and side effects; (5) Design: randomized controlled trial.

### Data extraction

Two authors independently extracted the following data: first author, year of publication, country, number of patients, patient characteristics, interventions, controls, adjuvant analgesics and main outcomes. We would contact the original authors by e-mail if data needed clarification or were not presented in the article. Extracted data were checked by the third author and any dispute was settled by consultation.

The primary outcome was pain score. Secondary outcomes included anxiety score, patient satisfaction and side effects. The pain score, anxiety score, and patient satisfaction were expressed using a visual analogue scale (VAS) or numeral rating scale (NRS) and was transformed to a 0–10 cm scale when the data were reported on a 0–100 mm scale. The data on the side effects were dichotomous. If the outcome data were not reported by mean±SD, they were converted according to the Cochran Handbook [18]. The definition of each outcome mentioned above was the same as that used in each included trial.

### Risk of bias and evidence grade assessment

Two authors independently assessed risk of bias in the included RCTs with the method recommended by the Cochrane Collaboration [19]. The quality of evidence for the outcomes were appraised using the Grading of Recommendations Assessment, Development and Evaluation (GRADE) approach [20]. Divergences were solved by discussion.

### Statistical analysis

The statistical analysis was conducted using Review Manager software (version 5.3; Nordic Cochrane Centre, Cochrane Collaboration). Differences were shown as the risk ratio (RR) with 95% confidence interval (CI) for dichotomous outcomes, and the weighted mean difference (MD) with 95% CI for continuous outcomes. The heterogeneity between the studies was assessed using I^2^. If I^2^ was<50%, the studies were considered as homogeneous, and a fixed-effect model was used. If I^2^ was ≥50%, there was significant heterogeneity among the studies, and a random-effects model was used. Potential sources of heterogeneity were identified by sensitivity analysis conducted by omitting one study in each turn and investigating the influence of a single study on the overall pooled estimate. Prior subgroup analyses were conducted to explore the influence of type of puncture biopsy, different controls, and gender on the overall pooled estimate. Publication bias was appraised by visually examining a funnel plot. P < 0.05 was considered statistically significant.

## Results

**Fig 1:**
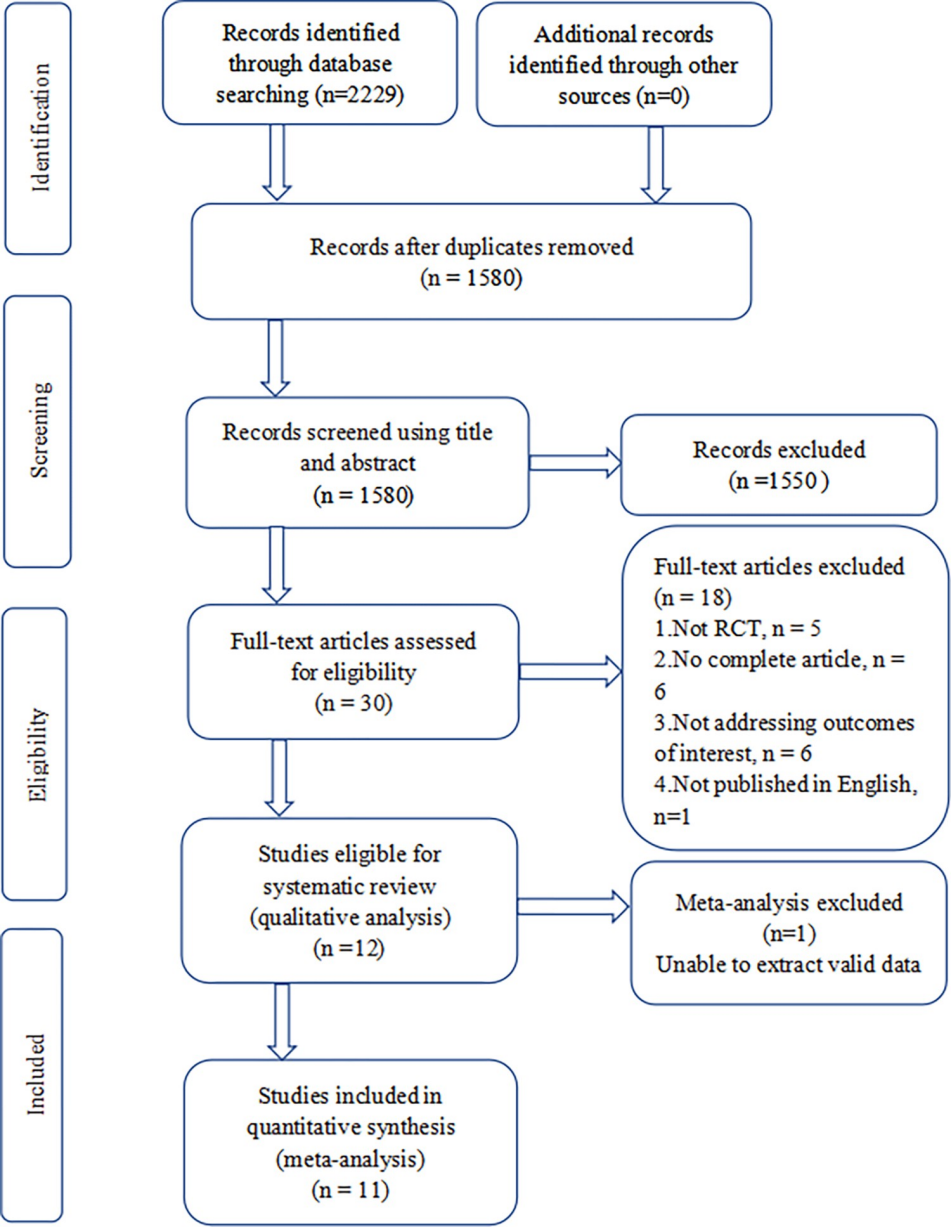
Flow chart of study selection.

The overall search yielded 2229 citations, of which 2199 were weeded out for various reasons based on the title and abstract. The full texts of the remaining 30 publications were scrutinized for further assessment and then twelve RCTs [21–32] were eligible for qualitative systematic review. One study [31] reported the results of the questionnaires completed by the physicians and patients regarding their degree of satisfaction with the examination process on a visual analog scale score, which was not consistent and could not been combined with the results of the other included RCTs. One study [32] reported the pain score as median and range and the data was converted as mean and SD in the pooled analysis [18, 33, 34]. Finally, eleven RCTs [21–30, 32] were included for meta-analysis (Fig 1).

### Study characteristics

The included RCTs were published between 2001 and 2022 with sample sizes ranging from 41 to 183. Among the included trials, 542 underwent experimental arm and 528 patients were in the control arm. Among the selected trials, ten [21–27, 29, 30, 32] were conducted in Europe, one [31] in Asia, and one [28] in South America. The participants in one trial [30] were only female, three [22, 23, 28] were only male, and the remaining eight [21, 24–27, 29, 31, 32] contained males and females. Two trials included three groups, of which one trial [22] included N_2_O group, air group and blank group, and the other trial [23] included N_2_O group, lidocaine group and blank group. One trial [30] compared N_2_O with 1% lidocaine in transabdominal chorionic villus sampling. One trial [32] compared N_2_O with midazolam in patients undergoing bone marrow biopsy. The remaining eight trials [21, 24–29, 31] compared N_2_O with placebo (Oxygen in five RCTs [21, 24, 26, 28, 31] and N_2_/O_2_ in three RCTs [25, 27, 29]. The diagnostic procedures included PLB [21, 25], TUSPB [22, 23, 28], bone marrow biopsy [24, 26, 32], LP [27, 29], transabdominal chorionic villus sampling [30] and endoscopic ultrasound-guided fine needle aspiration for digestive tract diseases [31]. The characteristics and outcomes of included trials were presented in Table 1.

**Table 1:** Characteristics of included randomized controlled trials.

		Participants							
Author	Country	No. of patients (N/C)	Gender (M/F)	Age (years)	Operation types	Intervention	Control	Adjuvant analgesics	Outcomes
Castéra et al. 2001 [21]	France	51/49	N group: 26/25; C group: 34/15	N group: 46 ± 13; C group: 42 ±11	Percutaneous liver biopsy	50% N_2_O/O_2_ inhalation 5 min before and during biopsy	Oxygen inhalation 5 min before and during biopsy	Local anesthesia with local infiltration of 10ml 1% xylocaine	Pain score (VAS: 0-100mm); Side effects; Cost
Masood et al. 2002 [22]	United Kingdom	51/45	All male	Not stated	Transrectal ultrasound-guided prostate biopsy	N_2_O/O_2_ inhalation 5 min before and during biopsy	Air inhalation	10 ml 2% rectal lignocaine hydrochloride local anesthetic gel	Pain score (VAS: 0-10cm)
Manikandan et al. 2003 [23]	United Kingdom	74/75	All male	N group: 65.21±8.3(43–87); C group: 64.96±8(51–83)	Transrectal ultrasound-guided prostate biopsy	Entonox inhalation 2 minutes before the procedure	1% lidocaine periprostatic infiltration	None	Pain score (VAS:0-10cm); Side effects
Johnson et al. 2007 [24]	United Kingdom	24/24	N group: 15/9; C group: 14/10	≥18	Bone marrow biopsy	50% N_2_O /O_2_ inhalation	Oxygen inhalation	Local anesthesia with 2% lignocaine	Pain score (VAS: 0-10cm); Willing to receive the same examination again
Meskine et al. 2011[25]	France	50/49	N group: 28/22; C group: 30/19	N group: 53.6 ±12.2; C group: 57.2 ±15.1	Percutaneous liver biopsy	N_2_O /O_2_ inhalation	50% N_2_/O_2_ inhalation	Local anesthesia with local infiltration of 10 ml 2% lidocaine	Pain score (VAS: 0-100mm); Satisfaction
Kuivalainen et al. 2015 [26]	Finland	35/35	N group: 16/19; C group: 21/14	N group: 58 ±13; C group: 60 ±12	Bone marrow biopsy	50% N_2_O /O_2_ inhalation	50% mixture of oxygen in air inhalation	Local anesthesia with local infiltration of 20 mg/ml plus epinephrine 5 μg/ml was infiltrated	Pain score(NRS: 0-10cm); Side effects
Moisset et al. 2016 [27]	France	33/33	N group: 19/14; C group: 17/16	N group: 44.7±10.8; C group: 42.4 ±11.6	Lumbar puncture	50% N_2_O /O_2_ inhalation	22% O_2_ /78% N_2_ inhalation	EMLA cream	Pain score (NRS: 0–10cm); Anxiety level (NRS); Satisfaction
Cazarim et al. 2018 [28]	Brazil	42/42	All male	N group: 69.45 ±8.42; C group: 66.38 ±7.19	Transrectal ultrasound-guided prostate biopsy	50% N_2_O /O_2_ inhalation	100% oxygen inhalation	Topical anesthesia in the anal canal with 2% lidocaine hydrochloride jelly	Pain score (VAS: 0–10); Side effects; Satisfaction
Nicot et al. 2022 [29]	France	44/44	N group: 16/27; C group: 18/23	N group: 37.3 ± 15.3; C group: 47.2 ± 20.0	Lumbar puncture	50% N_2_O /O_2_ inhalation	22% O_2_ /78% N_2_ inhalation	EMLA cream	Pain score (NRS: 0-10cm); Anxiety (NRS); Side effects; Satisfaction
Katsogiannou et al. 2018 [30]	France	93/90	All female	N group: 34.70 ± 5.21; C group: 34.27 ± 6.07	Transabdominal chorionic villus sampling	N_2_O /O_2_ inhalation	1% lidocaine Local anesthesia	None	Pain score (VAS: 0-10cm); Anxiety (VAS); Adverse events
Wang et al. 2016 [31]	China	21/20	25/16	42.4(47–69)	Endoscopic ultrasound-guided fine-needle aspiration for digestive tract diseases	30%-70% N_2_O /O2 inhalation	100% pure oxygen 2–3 L/min inhalation	None	Satisfaction; Willing to receive the same examination again; Side effects
Chakupurakal et al. 2008 [32]	United Kingdom	24/22	N group: 22/4; C group: 15/8	N group: 64(33–90); C group: 59(30–84)	Bone marrow biopsy	Entonox inhalation	Intravenous titrated midazolam 5–10 mg	Local anesthesia	Pain score; Side effects

Data are presented as mean± SD unless indicated otherwise.

Abbreviations: N group, Nitrous Oxide group; C group, Control group. EMLA cream, a eutectic mixture of local anesthetics containing lidocaine and prilocaine.

### Risk of bias assessment

**Fig 2:**
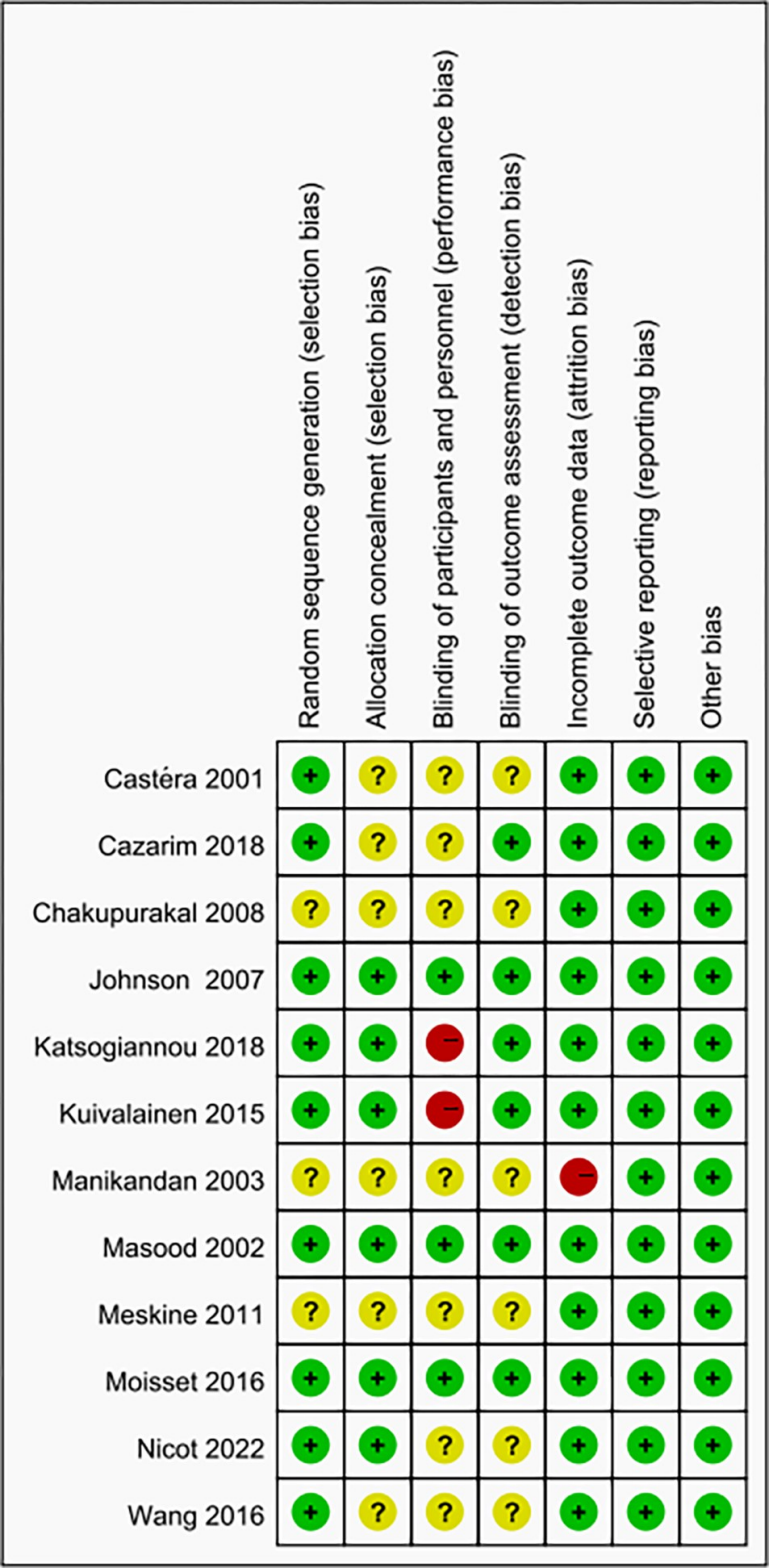
Risk of bias assessment.

Randomized sequence generation was fully described in nine trials [21, 22, 24, 26–31] and was judged to be unclear in three trials [23, 25, 32] as a result of not being reported. Allocation sequence concealment was adequately conducted in six trials [22, 24, 26, 27, 29, 30] through sequentially sealed envelopes or other hiding methods and the remaining six [21, 23, 25, 28, 31, 32] was judged to be unclear based on the available data. Blinding of participants and personnel was conducted in three RCTs [22, 24, 27] and was not conducted in two RCTs [26, 30]. The remaining seven RCTs [21, 23, 25, 28, 29, 31, 32] were judged to be unclear due to not being mentioned. The blinding of outcome assessment was described in six RCTs [22, 24, 26–28, 30] and not reported in six studies [21, 23, 25, 29, 31, 32]. The participants and reasons for withdrawal/ dropout were detailed reported in all RCTs except one [23]. None were defined as having selective reporting or other sources of bias across trials. Consequently, three trials [22, 24, 27] were defined as low risk of bias, and six trials [21, 25, 28, 29, 31, 32] were at unclear risk of bias, whereas three [23, 26, 30] were at high risk of bias. The risk of bias assessment for all included trials and details for judgment of bias was presented in Fig 2 and S1 Table, respectively.

### Primary outcome

**Fig 3:**
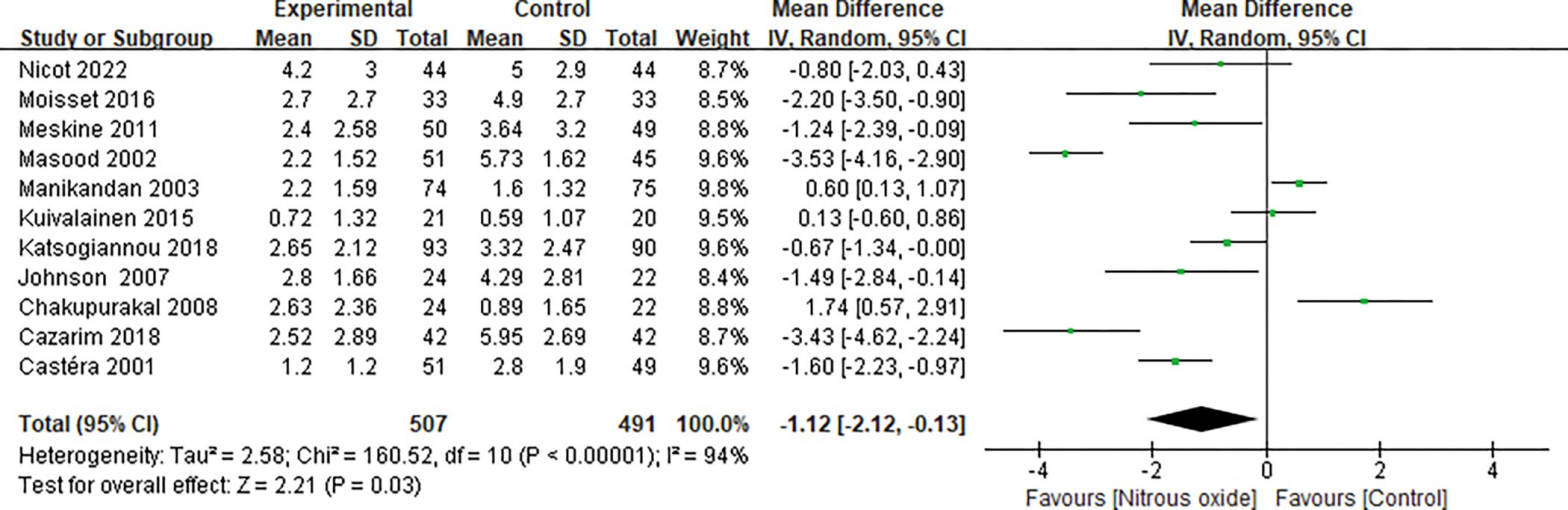
Forest plot for the pain score.

**Fig 4:**
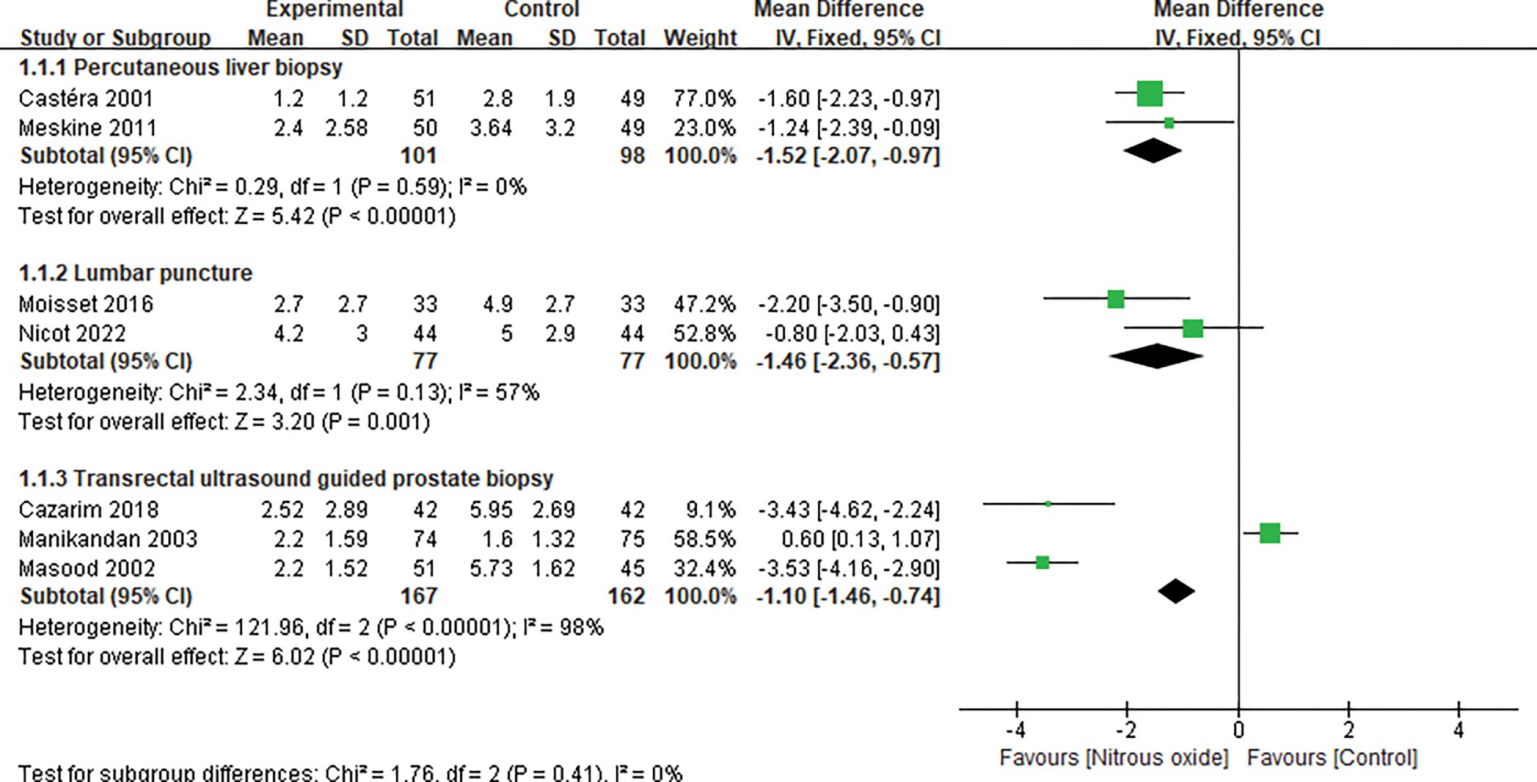
Forest plot of subgroup analysis for pain score by different operation types.

Data on pain score were available from eleven trials [21–30, 32]. Seven [21–25, 28, 30] measured the pain score by VAS, and four [26, 27, 29, 32] using NRS. Most of the trials assessed the outcome within half an hour after the operation was stopped. Pooled analysis suggested that N_2_O improved the pain score of puncture biopsy (MD -1.12, 95% CI -2.12 to -0.13, P = 0.03, Fig 3) with significant heterogeneity (I^2^ = 94%). Sensitivity analyses were performed by omitting one study in each turn and did not significantly alter the heterogeneity (P<0.0001, with I^2^ from 89 to 94%). Subgroup analyses were conducted to examine the influence of type of puncture biopsy, different controls and gender on the pain score. The superiority of N_2_O for painful diagnostic procedure was significantly evident in patients receiving PLB [21, 25] (MD -1.52, 95% CI -2.07 to -0.97, P<0.00001; I^2^ = 0%), LP [27, 29] (MD -1.46, 95% CI -2.36 to -0.57, P = 0.001; I^2^ = 57%), and TUSPB [22, 23, 28] (MD -1.10, 95% CI-1.46 to -0.74, P<0.00001; I^2^ = 98%) (Fig 4). Pooled analysis suggested that N_2_O could alleviate pain both in males’ [22, 23, 28] (MD -1.10, 95% -1.46 to -0.74, P<0.00001; I^2^ = 98%) and in females’ [30] (MD -0.67 95% -1.34 to -0.005) (S1 Fig). In addition, the pain score in the N_2_O group was significantly lower than that in the placebo group [21, 22, 24–29] (MD -1.77, 95% CI -2.79 to -0.75, P = 0.0006; I^2^ = 90%) and higher than that in the midazolam group [32] (MD 1.74, 95% CI 0.57 to 2.91, P = 0.004), while there was no significant difference between the N_2_O group and the lidocaine group [23, 30] (MD -0.01, 95% CI -1.26 to 1.23, P = 0.99; I^2^ = 89%) (S2 Fig).

### Secondary outcomes

**Fig 5:**
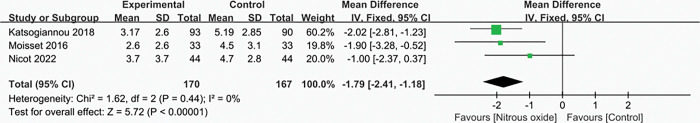
Forest plot for the anxiety score.

**Fig 6:**
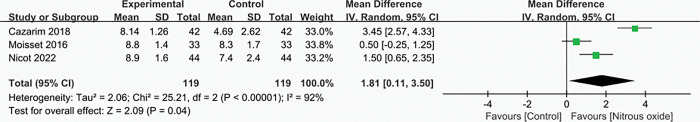
Forest plot for patient satisfaction.

Three trials [27, 29, 30] with 337 patients reported the anxiety score. Pooled analysis indicated that N_2_O significantly relieved patient anxiety (MD -1.79, 95% CI -2.41 to -1.18, P<0.00001; I^2^ = 0%, Fig 5). Three trials [27–29] with a total of 238 patients reported patient satisfaction. N_2_O significantly improved patient satisfaction (MD 1.81, 95%CI 0.11 to 3.50, P = 0.04; I^2^ = 92%, Fig 6).

### Side effects

Data for nausea and headache were available from three [21, 26, 29] and two [21, 26] RCTs, respectively. There was no significant difference in the incidence of nausea (RR 2.56; 95% CI 0.70 to 9.31, P = 0.15; I^2^ = 0%, S3 Fig) and headache (RR 0.62, 95% CI 0.17 to 2.33, P = 0.48; I^2^ = 46%, S4 Fig) between N_2_O group and the control group.

Dizziness was reported in two RCTs [26, 28]. Pooled analysis showed no significant difference between groups (RR 1.80, 95% CI 0.63 to 5.13, P = 0.27; I^2^ = 0%, S5 Fig). Data on euphoria was available in two trials [28, 29]. There was no significant difference between the two groups (RR 2.67, 95%CI 0.81 to 8.79, P = 0.11; I^2^ = 8%, S6 Fig).

### Quality of evidence and publication bias

The quality of evidence was low for pain score and moderate for the other remaining outcomes, which was shown in Table 2. As for publication bias of the pain score of N_2_O compared with the control group, visually inspecting funnel plots showed no evidence of potential publication bias among the included RCTs [35] (S7 Fig).

**Table 2:** Summary of GRADE evidence profile.

Outcome	No. of studies	Study design	Risk of bias	Inconsistency	Indirectness	Imprecision	Publication bias	Effect size	Certainty	Importance
								(95% CI)		
Pain score	9	RCT	Serious ^a^	Serious ^b^	Not Serious	Not Serious	Undetected	MD -1.73	Low	Critical
								(-2.54, 0.92)		
Anxiety score	3	RCT	Serious ^a^	Not Serious	Not Serious	Not Serious	Undetected	MD -1.79	Moderate	Important
								(-2.41, -1.18)		
Degree of satisfaction	3	RCT	Not Serious	Serious ^b^	Not Serious	Not Serious	Undetected	MD 1.81	Moderate	Important
								(0.11, 3.50)		
Incidence of nausea	3	RCT	Not Serious	Not Serious	Not Serious	Serious ^c^	Undetected	RR 2.56	Moderate	Important
								(0.70, 9.31)		
Incidence of headache	2	RCT	Not Serious	Not Serious	Not Serious	Serious ^c^	Undetected	RR 0.62	Moderate	Important
								(0.17, 2.33)		
Incidence of dizziness	2	RCT	Not Serious	Not Serious	Not Serious	Serious ^c^	Undetected	RR 1.80	Moderate	Important
								(0.63, 5.13)		
Incidence of euphoria	2	RCT	Not Serious	Not Serious	Not Serious	Serious ^c^	Undetected	RR 2.67	Moderate	Important
								(0.81, 8.79)		

Abbreviations: GRADE, quality of evidence grade; CI, confidence interval; RCT, randomized controlled trial; RR, risk ratio; SMD, standardized mean difference

a: Data reported as downgraded because of some concerns of bias.

b: Substantial heterogeneity (I^2^ = 88%) and (I^2^ = 87%) was found.

c: Data reported as downgraded because of wide CI or inadequate studies.

## Discussion

To our best knowledge, this was the first systematic review and meta-analysis on the efficacy and safety of N_2_O in adults undergoing puncture biopsy. The accumulated evidence from the present study showed that N_2_O might be an effective way of analgesia and sedation, which relieved pain perception and anxiety in patients undergoing diagnostic painful procedures. Additionally, compared with other analgesic methods, N_2_O had no excessive side effects.

To date, puncture biopsy is still a crucial method for the identification and analysis of pathological tissue and cell morphology to help doctors to make the pathological diagnoses. Generally speaking, conventional puncture biopsy is conducted when the patient is conscious. Pain, fear and discomfort with regard to the puncture biopsy often reduce patient’s compliance, which may delay the timely diagnosis and treatment of the tumor. Nowadays, a common analgesia mode is local anesthesia with lidocaine [36] in minor operations or invasive procedures like biopsies and minor excisions [37, 38]. However, many patients complain that local anesthesia does not provide enough analgesia during puncture biopsy. Eisenberg et al. [39] reported that 69% of patients felt pain immediately after the operation, and more than half of patients were still in pain four hours later. Several studies also suggested that local anesthesia fail to provide sufficient early and late analgesia [7–10]. Although Chakupurakal et al’s study [32] indicated that midazolam was superior to N_2_O in providing pain relief during bone marrow aspirate and trephine biopsy, midazolam caused respiratory depression, and care must be taken to monitor respiratory function which may prolong the length of hospital stay. Our pooled analysis indicated that compared with the controls, N_2_O had better analgesic effect with significant heterogeneity. The heterogeneity could be caused by several critical factors, such as the gender of patients, type of operation, control group, ratio of N_2_O/O_2_, inhalation time, the time point of data collection, and the number of puncture biopsies. Considering the limited number of studies included, the findings in the subgroup analyses should be evaluated rigorously and more high-quality RCTs are needed in the future.

N_2_O was a self-administered inhaled gas reserved in a pre-prepared cylinder and trained nurses can conduct without the presence of professional anesthesiologists. Its action is rapid and reversible after stopping inhalation and N_2_O has no depressive effects on the respiratory or cardiovascular function [40], and does not obscure the signs and symptoms that may be necessary for disease diagnosis. The mixture of 20% N_2_O and 80% oxygen has an equivalent analgesic effect as well as a good sedative effect with 15 mg morphine [41]. Three trials [27, 29, 30] reported the anxiety score. Pooled results suggested that N_2_O significantly reduced patients’ anxiety. Notably, Clark et al. [42] reported that N_2_O has amnesia characteristics so that patients often express an inability to recall severe pain or tension, even the entire procedure. In recent years, several studies have shown that N_2_O can be used in minor dental surgery, labor analgesia, pediatric operation pain (such as botulinum toxin injections, vaccine administration and intravenous catheter placement), and trauma first aid [43–46]. As for nausea, headache, dizziness and euphoria, pooled analysis showed that there was no significant difference between the N_2_O group and the control group. This finding may be related to the short inhalation time or low concentration of N_2_O. Most of the included studies applied 50% N_2_O/O_2_ inhalation 5 min before and during the biopsy, and the N_2_O/O_2_ inhalation was stopped at the end of the operation. In addditon, the frequency of side effects was relatively low among the included studies. Other side effects recorded in the included studies included oxygen desaturation (pulse oximetric saturation ≤94%), arterial hypotension, bradycardia, numbness and drowsiness, which usually disappeared rapidly after discontinuation [47]. N_2_O is a greenhouse gas and anesthesia is responsible for approximately 1% of N_2_O emissions in the atmosphere. Hence, N_2_O should be used by trained people and attention should be paid to scavenging it properly.

There were several limitations in the present study. Firstly, several included RCTs did not describe randomization and blinding in detail and the included trials were mostly single-blinded, which might affect the reliability of the pooled results. Secondly, the type of puncture biopsy and control groups varied among the included RCTs, which might lead to observed heterogeneity and thus impairing the robustness of our findings. Thirdly, several secondary outcomes and subgroup analysis only included not more than two RCTs, which need more large sample RCTs in the future so as to increase the reliability of the results. Fourthly, the outcome data in several included RCTs were not expressed as mean±SD. The measurement tools and time point of data collection also varied in the included studies. The data conversion and standardization were conducted in the pooled analysis, which might impair the robustness of the results. Finally, pain and anxiety perception were subjective outcomes and were not likely to be consistently assessed by individual patients.

## Conclusion

In summary, the present systematic review and meta-analysis suggested that the use of N_2_O might provide a better analgesic effect and slight side effects for puncture biopsy. However, due to the poor overall quality of the included studies and limited evidence, more well-designed randomized controlled trials are still needed to confirm our findings in the future.

## Supporting information

S1 Checklist PRISMA checklist. (DOCX)

S1 Table Support for judgment of bias. (DOCX)

S1 Fig Forest plot of subgroup analysis for pain score by gender. (TIF)

S2 Fig Forest plot of subgroup analysis for pain score by different control groups. (TIF)

S3 Fig Forest plot for nausea. (TIF)

S4 Fig Forest plot for headache. (TIF)

S5 Fig Forest plot for dizziness. (TIF)

S6 Fig Forest plot for euphoria. (TIF)

S7 Fig Funnel plot for pain score between the nitrous oxide group and the control group. (TIF)
