# Supplementary material for: Efficacy of nitrous oxide in adults undergoing puncture biopsy: A systematic review and meta-analysis of randomized controlled trials
Source: PLoS One. 2023 Jun 6;18(6):e0286713. doi: 10.1371/journal.pone.0286713 (PMC10243628; doi:10.1371/journal.pone.0286713)
Supplement: S1 Table — (DOCX) [file pone.0286713.s002.docx]

S1 Appendix. Support for judgment of bias.

| **Bais** | **Author**  **judgment** | **Support for**  **judgment** |
| --- | --- | --- |
| **Castéra et al ^2001^** | | |
| Random sequence generation (selection bias) | Low | Patients were randomized (using random numbers) to receive during biopsy a breathing mixture of equal parts of nitrous oxide and oxygen (N_2_O group) or a breathing oxygen placebo (P group). |
| Allocation concealment (selection bias) | Unclear | Not mentioned. |
| Blinding of participants and personnel (performance bias) | Unclear | Not mentioned. |
| Blinding of outcome assessment (detection bias) | Unclear | Not mentioned. |
| Incomplete outcome data (attrition bias) | Low | No one was lost. |
| Selective reporting (reporting bias) | Low | All relevant outcomes were described. |
| Other bias | Low | All contents are complete. |
| **Masood et al ^2002^** | | |
| Random sequence generation (selection bias) | Low | Randomization was performed using the method of preprepared cards in envelopes. |
| Allocation concealment (selection bias) | Low | The gas cylinders were covered and they were blinded to the gas used. |
| Blinding of participants and personnel (performance bias) | Low | Neither the investigator nor nurse assisting was involved in the randomization process since the gas cylinders were covered and they were blinded to the gas used. All patients involved were blinded to the gas that they inhaled. |
| Blinding of outcome assessment (detection bias) | Low | Neither the investigator nor nurse assisting was involved in the randomization process since the gas cylinders were covered and they were blinded to the gas used. |
| Incomplete outcome data (attrition bias) | Low | No one was lost. |
| Selective reporting (reporting bias) | Low | All relevant outcomes were described. |
| Other bias | Low | All contents are complete. |
| **Manikandan et al ^2003^** | | |
| Random sequence generation (selection bias) | Unclear | Randomized, not description. |
| Allocation concealment (selection bias) | Unclear | Not mentioned. |
| Blinding of participants and personnel (performance bias) | Unclear | Not mentioned. |
| Blinding of outcome assessment (detection bias) | Unclear | Not mentioned. |
| Incomplete outcome data (attrition bias) | High | One person in the control group was lost to follow-up. |
| Selective reporting (reporting bias) | Low | All relevant outcomes were described. |
| Other bias | Low | All contents are complete. |
| **Johnson et al ^200^*^7^*** | | |
| Random sequence generation (selection bias) | Low | The patients were randomized by means of remote telephone computer-generated randomization to receive either N_2_O/O_2_ or placebo (oxygen). |
| Allocation concealment (selection bias) | Low | The cylinders of oxygen and nitrous oxide were housed in a portable double cylinder case, which completely covered both cylinders. Identical tubing and demand valves labelled 1 and 2 were attached to the cylinders. |
| Blinding of participants and personnel (performance bias) | Low | Identical tubing and demand valves labelled 1 and 2 were attached to the cylinders. The gases were administered via identical mouth pieces. Thus, the identity of the gases was concealed to both patient and researcher. |
| Blinding of outcome assessment (detection bias) | Low | Identical tubing and demand valves labelled 1 and 2 were attached to the cylinders. The gases were administered via identical mouth pieces. Thus, the identity of the gases was concealed to both patient and researcher. |
| Incomplete outcome data (attrition bias) | Low | No one was lost. |
| Selective reporting (reporting bias) | Low | All relevant outcomes were described. |
| Other bias | Low | All contents are complete. |
| **Meskine et al ^2011^** | | |
| Random sequence generation (selection bias) | Unclear | Randomized, not description. |
| Allocation concealment (selection bias) | Unclear | Not mentioned. |
| Blinding of participants and personnel (performance bias) | Unclear | Double-blind, not description. |
| Blinding of outcome assessment (detection bias) | Unclear | Double-blind, not description. |
| Incomplete outcome data (attrition bias) | Low | No one was lost. |
| Selective reporting (reporting bias) | Low | All relevant outcomes were described. |
| Other bias | Low | All contents are complete. |
| **Kuivalainen et al ^2015^** | | |
| Random sequence generation (selection bias) | Low | The randomization was performed using sealed envelopes. |
| Allocation concealment (selection bias) | Low | The envelope was not opened until after the participant had given informed consent. |
| Blinding of participants and personnel (performance bias) | High | The patient was kept blinded regarding which gas was given until after the telephone interview on the following day. The performing physician and the assisting team were not blinded. |
| Blinding of outcome assessment (detection bias) | Low | Then the blinded research nurse conducted the second interview. Patients were asked to score the intensity of the pain experienced during the phases of BMAB on the NRS 0–10. |
| Incomplete outcome data (attrition bias) | Low | No one was lost. |
| Selective reporting (reporting bias) | Low | All relevant outcomes were described. |
| Other bias | Low | All contents are complete. |
| **Moisset et al ^2016^** | | |
| Random sequence generation (selection bias) | Low | Patients were assigned to treatment groups with a randomization list generated by an independent methodologist (BP). |
| Allocation concealment (selection bias) | Low | Placing the purchased gas bottles in a custom-built wooden box. |
| Blinding of participants and personnel (performance bias) | Low | Double-blinding was maintained by placing the purchased gas bottles in a custom-built wooden box. Perfumed masks were also used to disguise the smell of the product. |
| Blinding of outcome assessment (detection bias) | Low | Double-blinding was maintained by placing the purchased gas bottles in a custom-built wooden box. |
| Incomplete outcome data (attrition bias) | Low | No one was lost. |
| Selective reporting (reporting bias) | Low | All relevant outcomes were described. |
| Other bias | Low | All contents are complete. |
| **Wang et al ^2016^** | | |
| Random sequence generation (selection bias) | Low | The patients were divided into an experimental group and control group using a random-number table. |
| Allocation concealment (selection bias) | Unclear | Not mentioned. |
| Blinding of participants and personnel (performance bias) | Unclear | This was a single-blind study and the patients were unaware of the identity of the inhaled gas. But personnel not mentioned. |
| Blinding of outcome assessment (detection bias) | Unclear | Not mentioned. |
| Incomplete outcome data (attrition bias) | Low | No one was lost. |
| Selective reporting (reporting bias) | Low | All relevant outcomes were described. |
| Other bias | Low | Other contents are complete. |
| **Cazarim et al ^2018^** | | |
| Random sequence generation (selection bias) | Low | The patients were divided into two groups according to a sequence of random numbers generated electronically through the program GraphPad Prism1. |
| Allocation concealment (selection bias) | Unclear | Not mentioned. |
| Blinding of participants and personnel (performance bias) | Unclear | Double-blind, not description. |
| Blinding of outcome assessment (detection bias) | Low | An investigator who was not involved in the procedure presented and explained the 10 cm VAS to the patients, to evaluate their pain intensity during the procedure and the level of satisfaction with the administered treatment. |
| Incomplete outcome data (attrition bias) | Low | No one was lost. |
| Selective reporting (reporting bias) | Low | All relevant outcomes were described. |
| Other bias | Low | All contents are complete. |
| **Katsogiannou et al ^2018^** | | |
| Random sequence generation (selection bias) | Low | The randomization was made in blocks of 6. |
| Allocation concealment (selection bias) | Low | In sequentially numbered opaque envelopes. |
| Blinding of participants and personnel (performance bias) | High | Open-label trial. |
| Blinding of outcome assessment (detection bias) | Low | When evaluating pain and anxiety, the VAS score was determined by a clinical trial assistant blinded to the allocation of patients. |
| Incomplete outcome data (attrition bias) | Low | No one was lost. |
| Selective reporting (reporting bias) | Low | All relevant outcomes were described. |
| Other bias | Low | All contents are complete. |
| **Nicot et al ^2022^** | | |
| Random sequence generation (selection bias) | Low | A randomization list (random-size blocs) generated by an independent methodologist (BP). |
| Allocation concealment (selection bias) | Low | The detailed procedure was described in a previous study (gas bottles in identical boxes, a perfumed mask to make the sweet odor of N_2_O-O_2_ undiscernible). |
| Blinding of participants and personnel (performance bias) | Unclear | Double-blind, not description. |
| Blinding of outcome assessment (detection bias) | Unclear | Double-blind, not description. |
| Incomplete outcome data (attrition bias) | Low | No one was lost. |
| Selective reporting (reporting bias) | Low | All relevant outcomes were described. |
| Other bias | Low | All contents are complete. |
| **Chakupurakal et al ^2008^** | | |
| Random sequence generation (selection bias) | Unclear | Not mentioned. |
| Allocation concealment (selection bias) | Unclear | Not mentioned. |
| Blinding of participants and personnel (performance bias) | Unclear | Not mentioned. |
| Blinding of outcome assessment (detection bias) | Unclear | Not mentioned. |
| Incomplete outcome data (attrition bias) | Low | No one was lost. |
| Selective reporting (reporting bias) | Low | All relevant outcomes were described. |
| Other bias | Low | All contents are complete. |
