# Supplementary figures and images for: Efficacy of nitrous oxide in adults undergoing puncture biopsy: A systematic review and meta-analysis of randomized controlled trials
Source: PLoS One. 2023 Jun 6;18(6):e0286713. doi: 10.1371/journal.pone.0286713 (PMC10243628; doi:10.1371/journal.pone.0286713)

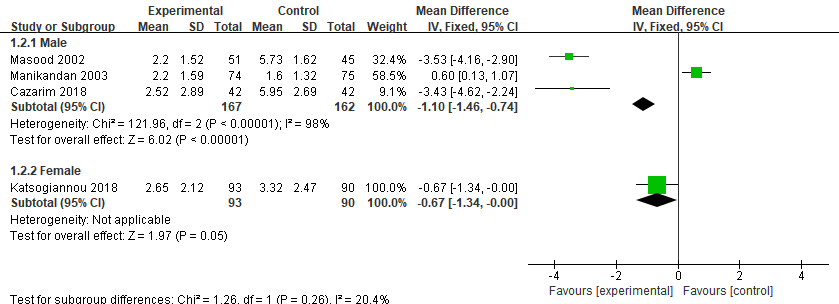

Supplement: S1 Fig — (TIF) [file pone.0286713.s003.tif]

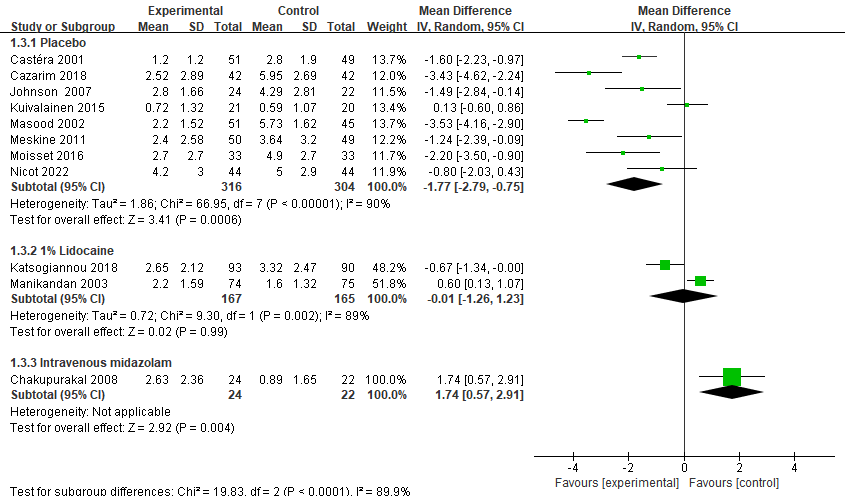

Supplement: S2 Fig — (TIF) [file pone.0286713.s004.tif]

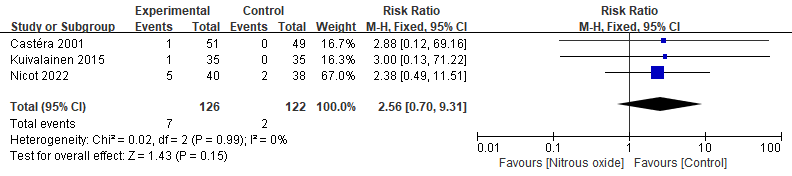

Supplement: S3 Fig — (TIF) [file pone.0286713.s005.tif]

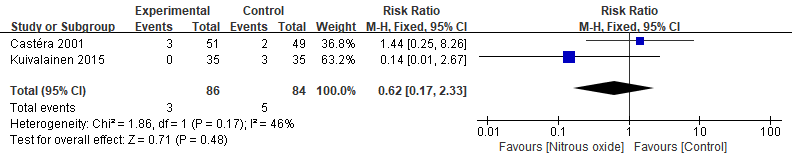

Supplement: S4 Fig — (TIF) [file pone.0286713.s006.tif]

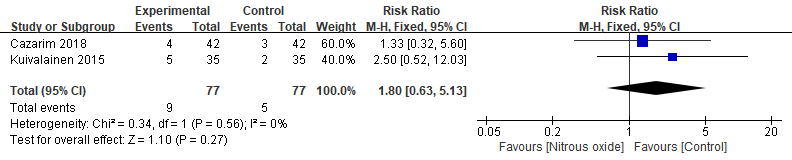

Supplement: S5 Fig — (TIF) [file pone.0286713.s007.tif]

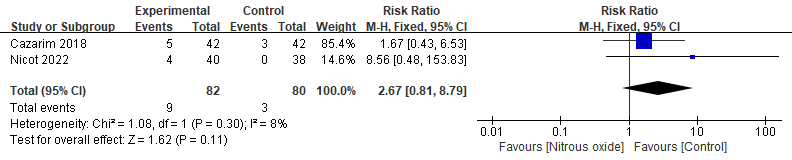

Supplement: S6 Fig — (TIF) [file pone.0286713.s008.tif]

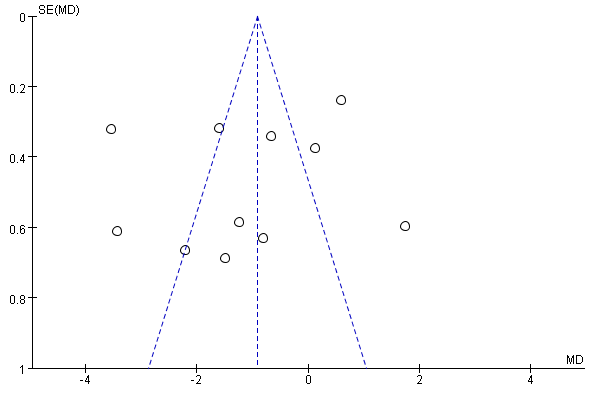

Supplement: S7 Fig — (TIF) [file pone.0286713.s009.tif]
